# Supplementary material for: Phenotypic Plasticity Index as a Strategy for Selecting Water-Stress-Adapted Coffee Genotypes
Source: Plants (Basel). 2023 Nov 30;12(23):4029. doi: 10.3390/plants12234029 (PMC10708338; doi:10.3390/plants12234029)
Supplement: Supplementary file 1 [file plants-12-04029-s001.zip › plants-2609515-supplementary.pdf]

Supplementary material

Table S1: Identification and genealogy of genotypes from the Minas Gerais Germplasm Collection (GC-MG).

Number	Identification	Genealogy
1	MG 270[1]	Hybrid Timor UFV 377-21
2	MG 270[2]	Hybrid Timor UFV 377-21
3	MG 364	Hybrid Timor UFV 442-42
4	MG 534	BE 5 Wush-Wush x Hybrid Timor UFV 366-08
5	MG 311	Hybrid Timor UFV 428-02
6	MG 279	Hybrid Timor UFV 376-31
7	MG 308	Hybrid Timor UFV 427- 55
8	Rubi MG1192	Catuaí and Mundo Novo
9	IPR 100	"Catuaí" x Coffee Plant ("Catuaí" x Coffee genotype from the 'BA-10' series) carrying genes from *C. liberica.*

[1] MG 270 block 1; [2] Selection of plants (1, 3, and 6) from the MG 270 accession in block 2.

Figure S1: Temperature (°C) and relative humidity (RH %) inside the greenhouse during the experimental period.

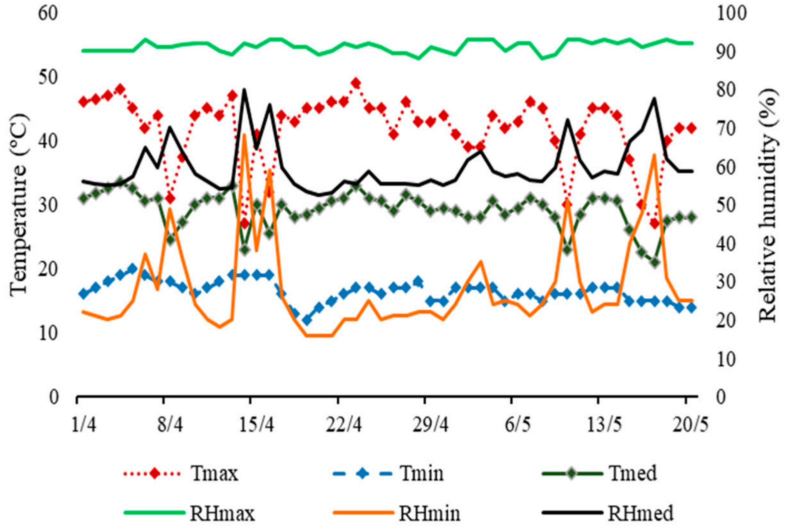

Figure S2: Graphical representation of climatological variables: maximum, mean, minimum temperatures, and precipitation recorded in the years 2019 (A) and 2020 (B).

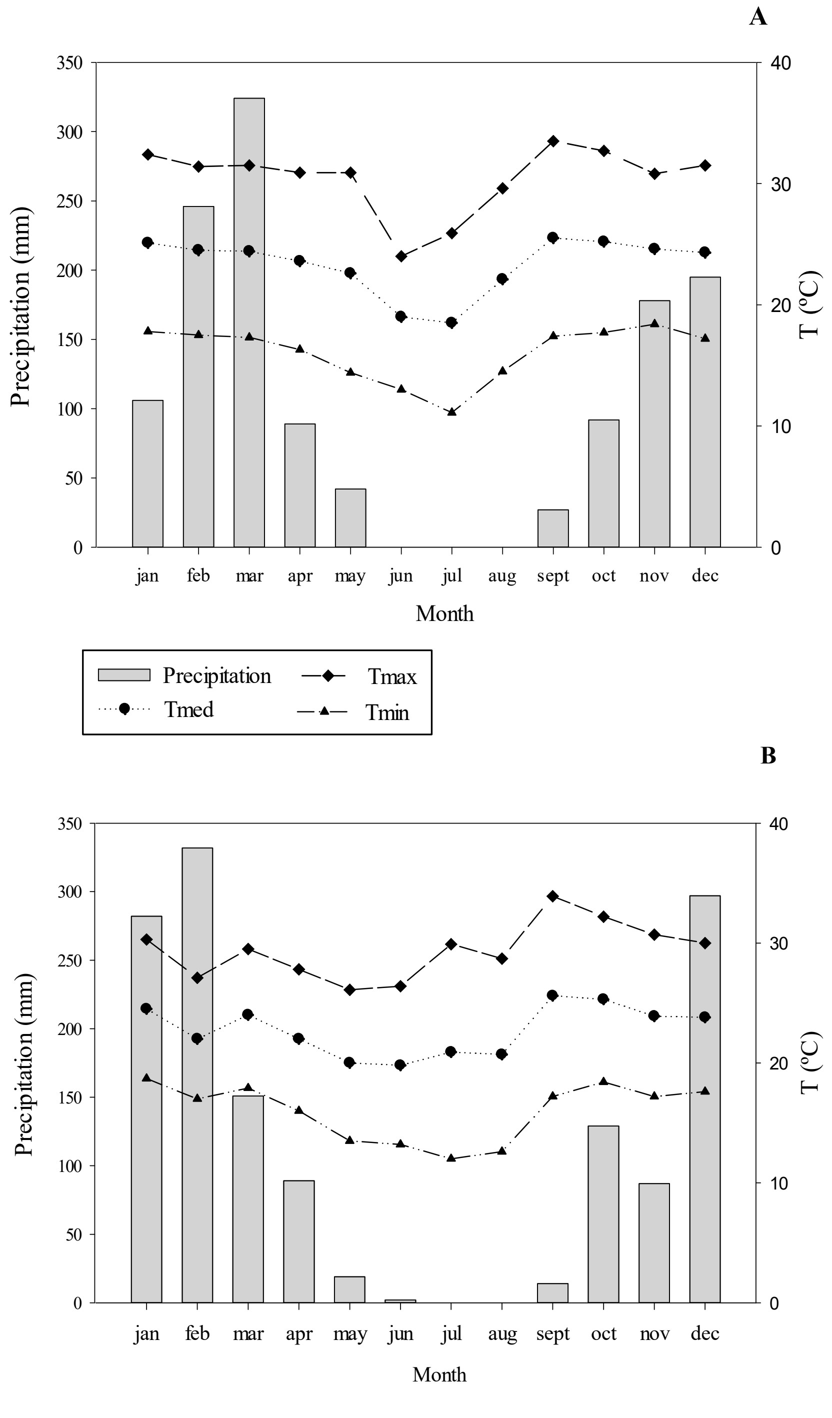

Source: Boa Vista Farm—DATERRA Agricultural Activities, Patrocínio-MG. (2020)

Figure S3: Mean values of net photosynthetic rate (a) and stomatal conductance (gs) of *Coffea arabica* L. genotypes under water deficit stress. A and D: Start of the experimental period; B and E: 25 days after water deficit imposition; C and F: genotype rehydration, 17 days after resuming irrigation.

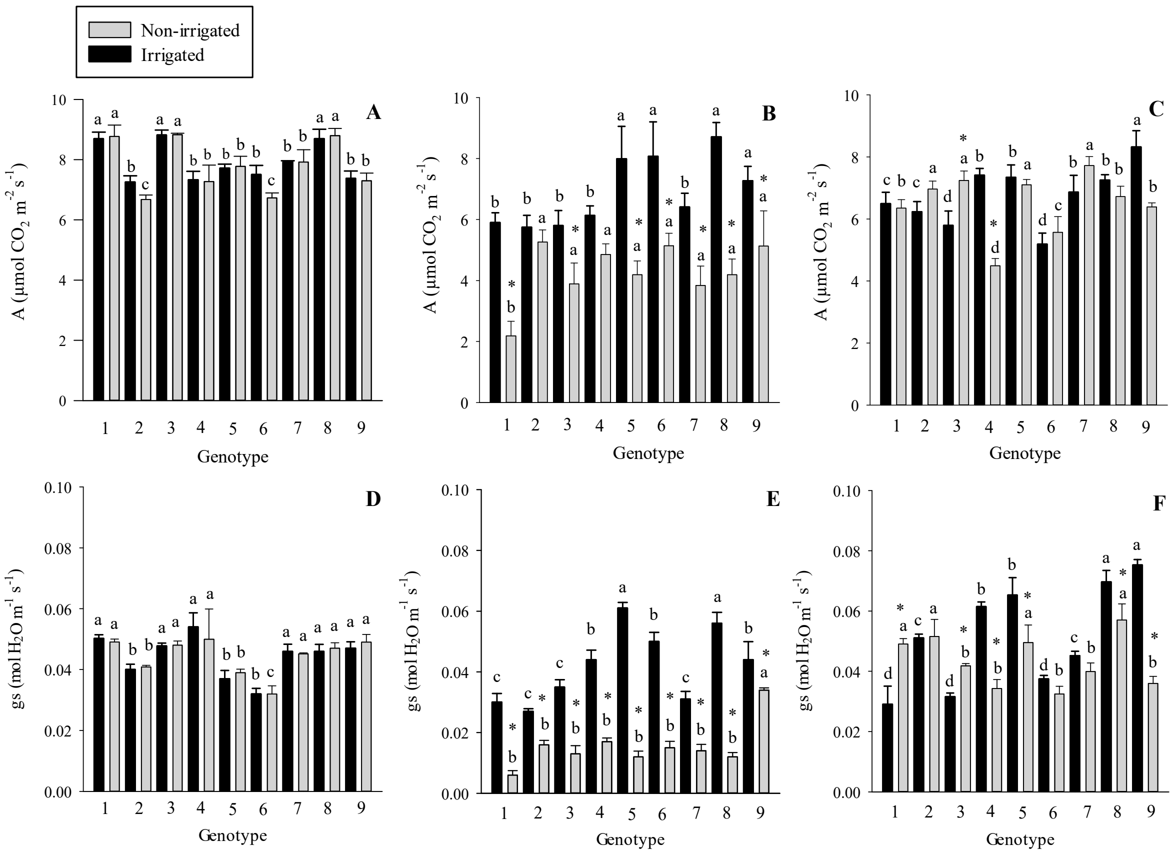

Means followed by the same lowercase letter compare genotypes within the water treatment, and * compares water treatments within each genotype, according to the Scott–Knott test at a 5% probability level.

Figure S4: Mean values of transpiration rate (E) and predawn water potential of *Coffea arabica* L. genotypes under water deficit stress. A and D: Start of experimental period; B and E: 25 days after water deficit imposition; C and F: genotype rehydration, 17 days after resuming irrigation.

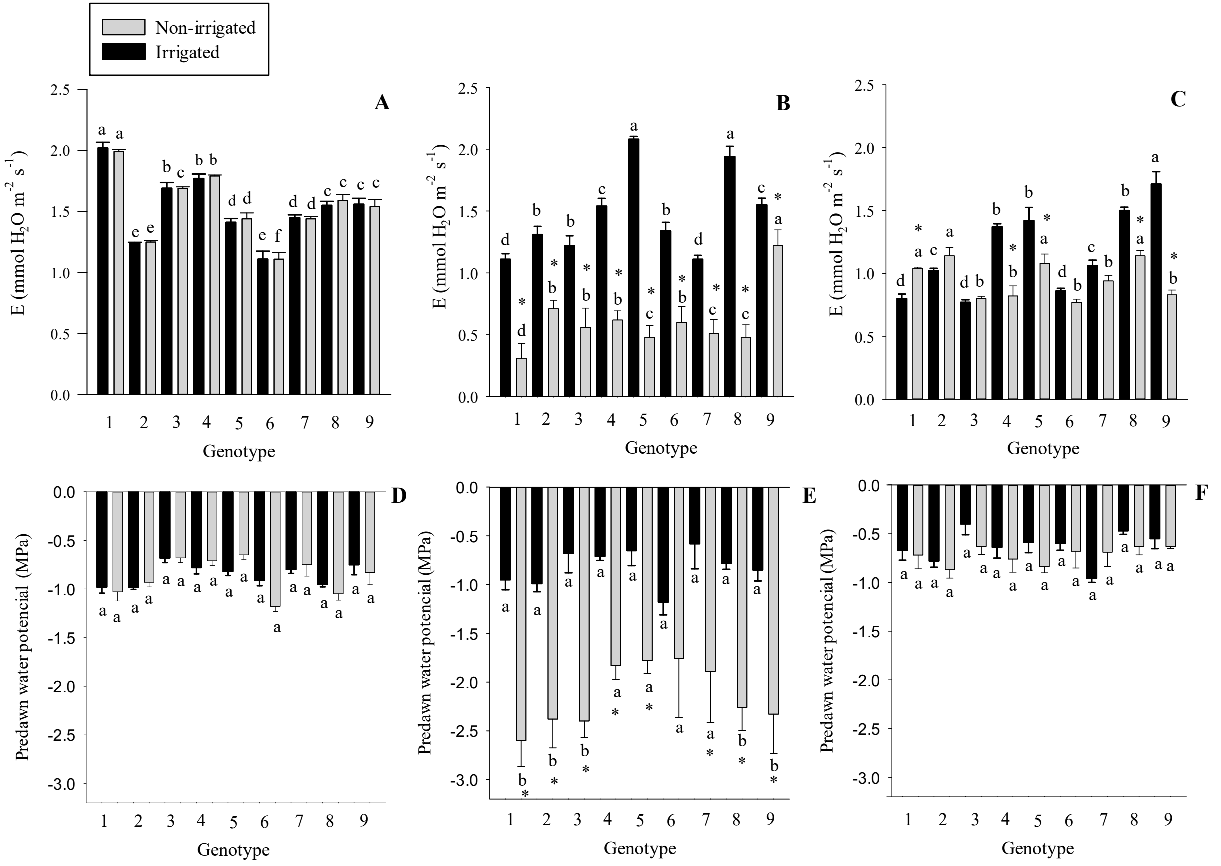

Means followed by the same lowercase letter compare genotypes within the water treatment, and * compares water treatments within each genotype, according to the Scott–Knott test at a 5% probability level.

Figure S5: Average instantaneous water use efficiency values of Coffea arabica L. genotypes subjected to water deficit. A - start of the experimental period; B - 25 days after the onset of water deficit imposition; C - rehydration of genotypes, 17 days after resumption of irrigation.

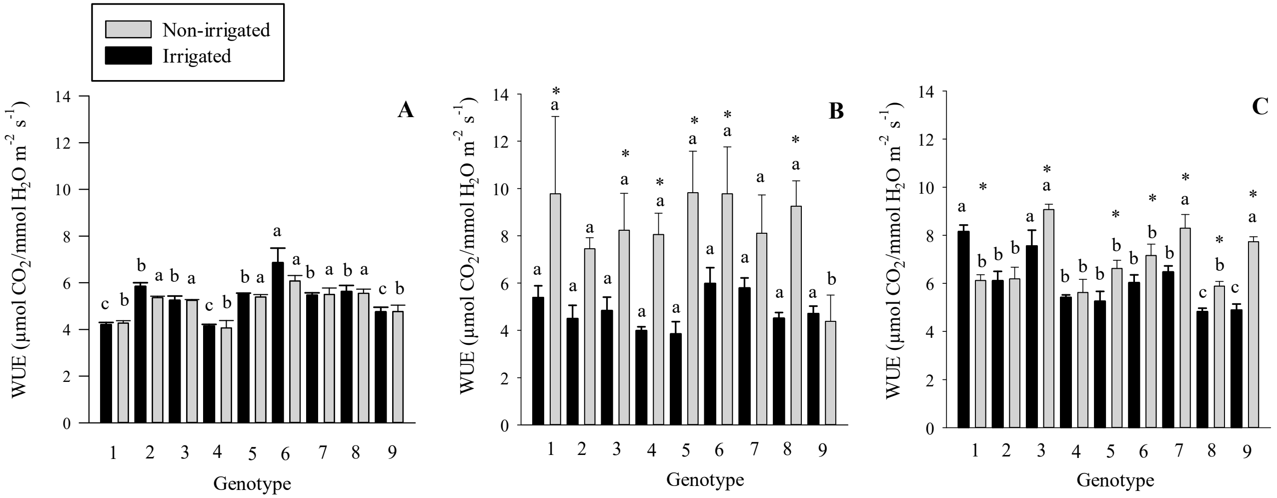

Means followed by the same lowercase letter compare genotypes within the water treatment, and * compares water treatments within each genotype, according to the Scott-Knott test at a 5% probability level.

Table S2: Hydrogen peroxide levels (H_2O_2 - mmol H_2O_2 g^{-1} FW) and malondialdehyde levels (MDA - mmol MDA g^{-1} FW) of *Coffea arabica* L. genotypes under water deficit stress in three different assessments[1].

Genotypes	H_2O_2			MDA		
	1	2	3	1	2	3
1	86.69 a	74.66 a	60.80 b	1.25 a	1.03 a	5.19 a
2	69.26 a	69.42 a	66.57 b	1.45 a	0.98 a	4.27 a
3	58.35 b	78.12 a	69.46 b	1.04 b	1.06 a	4.74 a
4	71.15 a	88.56 a	118.33 a	0.91 b	0.49 b	4.61 a
5	55.86 b	68.83 a	61.11 b	0.84 c	1.01 a	3.45 b
6	56.71 b	52.04 b	60.67 b	1.01 b	0.77 b	2.92 b
7	65.57 a	69.84 a	57.37 b	0.79 c	1.03 a	2.59 b
8	52.96 b	64.24 b	50.24 b	0.76 c	1.20 a	2.24 c
9	50.21 b	57.60 b	48.95 b	0.63 c	0.98 a	1.75 c

Means followed by the same letter in the column belong to the same group, according to the Scott–Knott grouping criterion, at a 5% probability level. ([1]) Assessments: 1—Start of experimental period; 2—25 days after water deficit imposition (DAWDI); 3—genotype rehydration, 17 days after resuming irrigation.

Table S3: Activity of catalase enzyme (CAT - μM H_2O_2 min^{-1} mg^{-1} FW) and superoxide dismutase enzyme (SOD - U SOD min^{-1} mg^{-1} FW) of *Coffea arabica* L. genotypes under water deficit stress in three different assessments[1].

Genotypes	CAT				SOD			
	1	2		3	1	2		3
		I	NI			I	NI	
1	0.011 c	0.009 b A	0.010 d A	0.004 b	0.47 b	0.61 b B	0.70 a A	0.41 b
2	0.013 c	0.004 b B	0.014 c A	0.005 b	0.48 a	0.74 a A	0.70 a A	0.43 a
3	0.020 b	0.007 b B	0.020 c A	0.009 a	0.49 a	0.76 a A	0.65 a B	0.43 a
4	0.022 b	0.008 b B	0.030 b A	0.011 a	0.49 a	0.72 a A	0.68 a A	0.45 a
5	0.014 c	0.006 b A	0.009 d A	0.011 a	0.49 a	0.69 a A	0.65 a A	0.45 a
6	0.023 b	0.007 b B	0.015 c A	0.005 b	0.48 b	0.67 b A	0.72 a A	0.41 b
7	0.021 b	0.016 a B	0.031 b A	0.013 a	0.47 b	0.63 b A	0.52 c B	0.38 b
8	0.018 b	0.009 b B	0.016 c A	0.006 b	0.47 b	0.59 b A	0.62 b A	0.38 b
9	0.030 a	0.017 a B	0.039 a A	0.008 a	0.45 c	0.62 b A	0.60 b A	0.38 b

Means followed by the same lowercase letter in the column and uppercase letter in the row belong to the same group, according to Scott-Knott grouping criterion, at a 5% probability level. ([1]) Assessments: 1—Start of the experimental period; 2—25 days after water deficit imposition (DAWDI); 3—genotype rehydration, 17 days after resuming irrigation. I—irrigated; NI—non-irrigated.

Table S4: Catalase enzyme activity (CAT - μM H_2O_2 min^{-1} mg^{-1} FW) relative to water treatment, 17 days after resuming irrigation.

TH	CAT
I	0.006 b
NI	0.010 a

TH:—water treatment; I—irrigated; NI—non-irrigated. Means followed by the same letter belong to the same group, according to the Scott–Knott grouping criterion, at a 5% probability level.

Table S5: Ascorbate peroxidase activity (APX - μM AsA min^{-1} mg^{-1} FW) and ascorbate levels (AsA - mg AsA g^{-1} FW) in three different assessments[1].

Genotypes	APX			AsA			
	1	2	3	1	2		3
					I	NI	
1	0.27 b	0.15 c	0.12 b	41.93 a	33.46 a B	54.68 a A	39.12 b
2	0.46 a	0.29 b	0.19 b	45.25 a	32.93 a B	50.74 a A	47.97 a
3	0.48 a	0.40 a	0.35 a	45.44 a	31.75 a B	42.42 b A	40.53 b
4	0.42 a	0.30 b	0.32 a	50.02 a	34.16 a A	39.40 b A	33.07 c
5	0.47 a	0.17 c	0.31 a	51.63 a	25.31 a B	37.83 b A	30.38 c
6	0.47 a	0.35 b	0.29 a	53.27 a	30.74 a A	35.11 b A	39.10 b
7	0.45 a	0.45 a	0.11 b	54.31 a	39.40 a A	25.37 c B	33.33 c
8	0.58 a	0.54 a	0.11 b	55.10 a	34.92 a B	44.63 a A	34.23 c
9	0.56 a	0.50 a	0.15 b	60.05 a	36.60 a A	40.36 b A	27.87 c

Means followed by the same lowercase letter in the column and uppercase letter in the row belong to the same group, according to Scott–Knott grouping criterion, at a 5% probability level. ([1]) Assessments: 1—start of experimental period; 2—25 days after water deficit imposition (DAWDI); 3—genotype rehydration, 17 days after resuming irrigation. I—irrigated; NI—non-irrigated.

Table S6: Ascorbate peroxidase activity (APX - μM AsA min^{-1} mg^{-1} FW) in the second and third assessment[1] relative to water treatment.

TH	2	3
I	0.17 b	0.31 b
NI	0.26 a	0.40 a

TH—water treatment; I—irrigated; NI—non-irrigated. Means followed by the same letter belong to the same group, according to the Scott–Knott grouping criterion, at a 5% probability level. ([1]) Assessments: 2—25 days after water deficit imposition (DAWDI); 3—genotype rehydration, 17 days after resuming irrigation.
